# Supplementary material for: Ovarian Accumulation of Nanoemulsions: Impact of Mice Age and Particle Size
Source: Int J Mol Sci. 2021 Jul 31;22(15):8283. doi: 10.3390/ijms22158283 (PMC8347032; doi:10.3390/ijms22158283)
Supplement: Supplementary file 1 [file ijms-22-08283-s001.zip › ijms-1255117-supplementary.pdf]

International Journal of
Molecular Sciences

MDPI

Supplementary Material

Ovarian accumulation of nanoemulsions: impact of mice age and particle size

Eike Folker Busmann, Julia Kollan, Karsten Mäder and Henrike Lucas

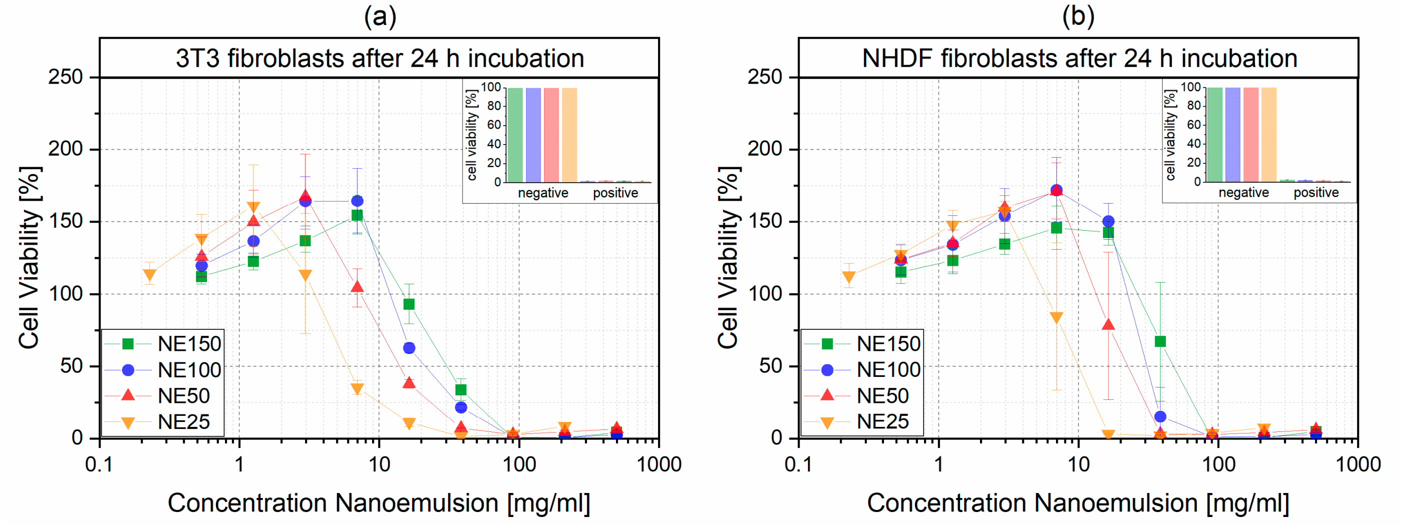

Figure S1. Dose-response curves of the cell viability over the total mass nanoemulsion (DiR loaded MCT, Kolliphor® HS 15 + aqueous phase) per ml cell culture media on (a) 3T3 and (b) NHDF fibroblasts after 24 h of incubation, determined by three individual incubated batches (eight replicates per run); the inlet graphs show the corresponding negative and positive controls with the untreated or Triton™ X-100 treated cells, respectively.

Table S1. Mean PARE values plus standard deviation (n = 5) of each excised organ plus withdrawn blood of the juvenile (juv.), adult (ad.), and senescent (sen.) mice i.v. injected with NE25, NE50, NE100, or NE150 nanoemulsions.

**Mean PARE [%] of the Excised Organs**	**juv. NE50**	**juv. NE100**	**juv. NE150**	**ad. NE25**	**ad. NE50**	**ad. NE100**	**ad. NE150**	**sen. NE25**	**sen. NE50**	**sen. NE100**	**sen. NE150**
blood	0.4 ± 0.1	0.6 ± 0.2	0.5 ± 0.2	0.3 ± 0.1	0.4 ± 0.1	0.8 ± 0.2	0.9 ± 0.3	0.5 ± 0.2	0.7 ± 0.2	1.0 ± 0.3	1.0 ± 0.2
heart	3.0 ± 0.3	4.0 ± 1.0	6.7 ± 1.3	2.1 ± 0.5	2.1 ± 0.4	4.1 ± 0.8	5.9 ± 0.6	2.1 ± 0.2	3.2 ± 0.6	3.8 ± 0.3	6.3 ± 1.1
lungs	1.8 ± 0.3	2.4 ± 1.9	2.2 ± 0.4	2.6 ± 0.4	1.6 ± 0.2	1.8 ± 0.3	2.1 ± 0.3	2.0 ± 0.1	2.0 ± 0.5	1.9 ± 0.3	1.9 ± 0.2
liver	65.6 ± 2.0	65.2 ± 7.1	60.8 ± 3.0	66.2 ± 1.6	62.8 ± 2.6	46.4 ± 4.7	40.3 ± 3.6	65.9 ± 3.4	58.0 ± 5.8	47.6 ± 3.8	42.8 ± 2.6
kidneys	2.5 ± 0.2	2.9 ± 1.0	3.2 ± 0.4	2.4 ± 0.3	3.0 ± 0.6	4.2 ± 0.4	4.6 ± 0.2	2.6 ± 0.2	3.9 ± 0.6	4.5 ± 0.5	6.2 ± 0.5
bladder	0.6 ± 0.1	0.7 ± 0.3	0.7 ± 0.2	0.6 ± 0.1	0.7 ± 0.2	1.2 ± 0.2	1.1 ± 0.1	0.6 ± 0.1	0.8 ± 0.2	0.9 ± 0.2	1.3 ± 0.5
spleen	9.5 ± 0.9	8.3 ± 3.3	10.8 ± 1.4	6.7 ± 0.8	11.4 ± 1.0	12.9 ± 1.2	8.3 ± 0.9	7.3 ± 0.9	7.6 ± 1.6	8.8 ± 0.9	8.6 ± 1.4
pancreas + fat	1.1 ± 0.1	1.1 ± 0.3	1.4 ± 0.3	0.8 ± 0.2	0.9 ± 0.1	1.4 ± 0.3	1.7 ± 0.2	0.9 ± 0.1	1.5 ± 0.2	1.6 ± 0.3	1.7 ± 0.4
stomach	2.3 ± 0.4	1.9 ± 0.9	2.1 ± 0.3	2.0 ± 0.6	2.0 ± 0.5	4.3 ± 0.5	6.4 ± 1.2	2.1 ± 0.4	3.4 ± 0.9	4.5 ± 0.8	6.4 ± 1.1
duodenum	0.9 ± 0.2	0.9 ± 0.3	0.9 ± 0.2	1.4 ± 0.2	1.2 ± 0.3	1.7 ± 0.3	2.1 ± 0.4	1.4 ± 0.4	1.8 ± 0.7	2.1 ± 0.3	2.2 ± 0.7
caecum	2.0 ± 0.3	2.0 ± 1.0	1.6 ± 0.6	1.4 ± 0.6	1.7 ± 0.4	3.1 ± 1.1	4.7 ± 0.3	1.7 ± 0.4	2.5 ± 0.7	3.7 ± 1.1	5.2 ± 0.9
colon	0.4 ± 0.1	0.6 ± 0.1	0.4 ± 0.1	0.6 ± 0.2	0.5 ± 0.2	1.6 ± 1.6	1.0 ± 0.1	0.4 ± 0.1	0.8 ± 0.1	0.8 ± 0.1	1.0 ± 0.3
brain	0.3 ± 0.1	0.3 ±0.0	0.3 ± 0.0	0.5 ± 0.0	0.4 ± 0.1	0.6 ± 0.1	0.8 ± 0.1	0.8 ± 0.1	1.1 ± 0.2	0.9 ± 0.1	1.1 ± 0.1

skin	1.4 ± 0.5	1.5 ± 0.4	1.4 ± 0.3	0.8 ± 0.1	1.6 ± 0.2	2.5 ± 0.8	3.3 ± 0.7	1.2 ± 0.4	2.0 ± 0.6	3.0 ± 1.2	3.4 ± 0.6
s.c. fat	1.2 ± 0.4	1.0 ± 0.2	1.3 ± 0.2	1.6 ± 1.3	1.2 ± 0.3	1.5 ± 0.4	2.3 ± 0.3	1.9 ± 2.0	2.0 ± 1.3	2.2 ± 0.3	1.9 ± 0.3
femur, knee + tibia	4.4 ± 0.6	3.9 ± 0.4	3.5 ± 0.5	3.4 ± 0.8	4.3 ± 0.6	4.6 ± 0.5	3.3 ± 0.4	3.4 ± 0.5	3.8 ± 0.9	3.7 ± 0.5	3.0 ± 0.4
thigh muscle	0.9 ± 0.3	0.9 ± 0.4	0.9 ± 0.2	1.5 ± 1.2	0.6 ± 0.1	1.2 ± 0.3	1.4 ± 0.2	0.8 ± 0.2	1.0 ± 0.3	1.2 ± 0.3	1.7 ± 0.7
uterus + ovaries	1.6 ± 0.8	1.7 ± 0.7	1.2 ± 0.1	5.2 ± 1.6	3.5 ± 0.4	5.9 ± 2.8	9.8 ± 2.3	4.3 ± 2.5	3.9 ± 1.5	7.6 ± 2.4	4.3 ± 1.5
